# Halloysite Nanotubes as an Additive to Ensure Enhanced Characteristics of Cold-Curing Epoxy Resins under Fire Conditions

**DOI:** 10.3390/polym12091881

**Published:** 2020-08-20

**Authors:** Jaroslav Hornak, Petr Kadlec, Radek Polanský

**Affiliations:** Regional Innovation Centre for Electrical Engineering, Faculty of Electrical Engineering, University of West Bohemia, Univerzitní 8, 301 00 Pilsen, Czech Republic; kadlecp6@ket.zcu.cz (P.K.); rpolansk@ket.zcu.cz (R.P.)

**Keywords:** dielectric properties, epoxy-based composites, fire retardancy, halloysite nanotubes, mechanical properties, thermal properties

## Abstract

At present, the most commonly used electrical insulating materials, including cold-curing epoxy resins, are well designed for normal operating conditions. However, new generations of materials should also be capable of withstanding extreme emergency conditions, e.g., in case of fire. For this reason, this study presents the possibilities of an improved cold-curing epoxy resin using halloysite nanotubes (HNTs) to increase its operational safety. The positive effect of HNT addition is indicated mainly in terms of the suppression of thermo-oxidation processes, which has been demonstrated by the decreases in the maximum heat flow peaks as well as the specific enthalpy values during the thermal decomposition of the epoxy resin. The observed dielectric parameters of the HNT-added materials differ only slightly from those without a filler, whereas their mechanical properties strongly depend on the amount of dispersed HNTs.

## 1. Introduction

Epoxy resins are one of the most widely used thermosetting plastics in electrotechnical applications [1], and they are mainly used due to beneficial properties such as their low shrinkage during curing, flexibility, strong adhesion to different substrates, chemical resistance, and excellent electrical insulating properties [2]. The most common products based on epoxy resins are currently adhesives, laminates, coatings, and sealants [3,4], all of which are widely used in various industries. As far as electrical engineering is concerned, epoxy resins are mainly used as the main wall insulation of rotating machines (mostly glass-fiber reinforced [5]) or as encapsulating and potting materials in electronics [6]. Since some electronic components are temperature sensitive, conventional potting materials cured at elevated temperatures cannot be used, and it is necessary to protect them by using cold-curing epoxy resins [7]. These resins are cured at ambient temperature, and the possibility of curing without additional equipment (curing oven) is also advantageous.

In addition to the aforementioned favorable properties, epoxy resins also have some general drawbacks that should be considered before use in potential applications. The most important factors are low impact strength, low resistance toward crack initiation, and relatively high flammability [8,9]. The limiting parameter for the use of cold-curing epoxy resins is mostly the low glass transition temperature [10]. Nevertheless, the dielectric and most of the mechanical properties of encapsulating and potting materials for electronics are now at their maximum values; hence, there are few ways for further improvement. However, there are still opportunities to reduce their flammability. This undesirable property may be partially suppressed using various micro- or nanosized particle fillers, assuming that the electrical and mechanical properties are not significantly impaired.

Among the promising particle fillers that can be used to modify polymer materials, including epoxy resins, is halloysite in a tubular form, which is often called halloysite nanotubes (HNTs) [11]. Halloysite (Si${}_{2}$Al${}_{2}$O${}_{2}$(OH)${}_{2}\cdot$2H${}_{2}$O) is representative of clay minerals (phyllosilicates) [12]. Specifically, it is a dioctahedral hydrated polymorph of kaolinite with a monolayer of water molecules placed between aluminosilicate layers [13]. Churchman et al. mention a steep increase in interest in Halloysite and its use in various areas [14]. In addition to the filling of polymeric materials [15], it is possible to use HNTs, for example, in the medicine [16], cosmetics [17], or agriculture [18]. In comparison with kaolin or montmorillonite, HNTs can be relatively easily dispersed in water or polar polymers due to their tubular morphology, charge distribution, and unique crystal structure [19] and thus form a suitable substance, inter alia, for biopolymer materials [20]. A general literature search focused on the polymer materials with HNTs led to the finding, that a considerable number of studies dealing with the thermal properties of polymer/HNTs composites have already been published [21,22,23,24]. Some studies [25,26] also emphasized a positive effect on fire resistance of epoxy-based materials. Especially in this case, the flammability of encapsulating and potting materials plays a significant role and must be strictly classified according to a number of standards, e.g., standards for flammability of plastic materials UL 94 [27]).

An improvement in the fire resistance of polymer materials has been an essential topic for decades. However, the health hazards of many chemicals used for such purposes have not been solved for a long time. After proving the actual hazards of some compounds, especially bromine- and chlorine-based compounds, these compounds have been prohibited in many countries (e.g., in the European Union based on a series of regulations and directives). Strict restrictions on the use of selected fire retardants are also based on information summarized by the European Chemicals Agency under REACH (Registration, Evaluation, Authorization, and Restriction of Chemicals). Safety precautions, as defined in RoHS (Restriction of Hazardous Substances) and other directives, were passed in this manner. The group of prohibited fire retardants includes polybrominated diphenyl ethers (PBDE) and polybrominated biphenyls (PBB). Natural mineral materials, such as clay minerals, which include HNTs, are one of the possible alternatives to these prohibited substances. Further information on these restrictions is given in more detail in [28,29,30,31].

Increased fire resistance is most often achieved by a chemical modification of the polymer [32], but as has already been mentioned, a suitable filler may also be used. Due to this modification, it is possible to achieve increased fire retardation and increased thermal conductivity while still working with an environmentally friendly and easily obtainable natural material.

For the reasons mentioned above, the main aim of this study is to describe the effect of HNTs addition on the thermal, dielectric and mechanical properties of cold-curing epoxy-based composites (EP/HNTs). These properties were comprehensively studied to provide insight into the behavior of EP/HNTs during thermal decomposition, which may be mainly useful in electrical engineering, where these materials are used for encapsulating and potting electronic components. However, the obtained results are also promising for other applications in which the flammability of epoxy resins needs to be controlled. The analysis of dielectric properties, including the frequency-temperature dependencies of dielectric parameters obtained by broadband dielectric spectroscopy for this type of composite, has not yet been discussed in detail in journals and may be important for furthering research on cold-curing epoxy-based materials.

## 2. Materials and Sample Preparation

All tested materials contain a two-component cold-curing epoxy resin, which is cured at ambient temperature. A series of tested materials include epoxy resin without filler additives (EP resin) and three groups of composites manufactured from the EP resin and HNT filler using different weight percentages (2.5, 6.5 and 10 wt% HNTs). Manufactured composites will be henceforth referred to as “EP/2.5HNT”, where 2.5 means the HNT weight percentage.

### 2.1. Composite Matrix

Elan-tech two-component epoxy resin (ELANTAS, Italy) was used for this study as a thermoset matrix. The first component (EC 141 NF) is a mixture of several chemical substances, of which the substance with a CAS No. of 25068-38-6 (4,4${}^{\prime }$-isopropylidenediphenol, oligomeric reaction products with 1-chloro-2,3-epoxypropane) is the most important. It is essentially an epoxy resin based on bisphenol A and epichlorohydrin with an average molar mass ≤700 g/mol [33]. The second component (W 241 NF) is a hardener, which ensures the formation of a cross-linked structure primarily due to the presence of 3-aminoethyl-3,5,5-trimethylcyclohexylamine (CAS No. 2855-13-2, also called isophoronediamine or polyamine). The first component was mixed with the second component in a weight ratio of 100:45. The used epoxy resin is a cold-curing resin [7] in which an elevated temperature is not required for curing; therefore, the resin was cured at an ambient temperature ranging from 22 to 25 ${}^{\circ }$C.

### 2.2. Clay Tubular Filler

Commercially available Dragonite HP (Applied Minerals, USA) clay filler, which is mined from the Dragon Mine in Utah, USA [34], was used for this study. The manufacturer reports that the HNT content in Dragonite is greater than 95% and that 89.9% of particles in Dragonite are smaller than 10 µm and 49.4% of particles are smaller than 0.2 µm [34]. However, the measurements on Dragonite (from the same mine), which have been published in an earlier study [35], indicate that Dragonite contains only 84% HNTs, and the rest of the clay is kaolinite, quartz, and other minerals. According to other information available from a previous study by Polanský et al. [36], the following properties, determined in delivered samples of Dragonite, can be assumed: Specific surface area *S_BET_* = 45.32 ± 1.38 m${}^{2}$·g${}^{-1}$, total pore volume *V_p_* = 0.165 ± 0.033 cm${}^{3}$·g${}^{-1}$ and zeta potential ζ = −4.1 ± 0.4 mV.

### 2.3. Test Samples Preparation

During the preparation of test samples, 15 g of EP resin (EC 141 NF) was first weighed and heated to 60 ${}^{\circ }$C to increase miscibility with the filler. Prior to mixing with EP resin, HNTs were dried at 60 ${}^{\circ }$C for 24 h to reduce the amount of absorbed moisture. Then, the HNTs were added to the EP resin in a weight percentage that corresponded to the desired filler concentrations (2.5, 6.5, and 10 wt% HNTs). The EP resin was mixed with the HNTs for 90 min with a laboratory magnetic stirrer at 800 rpm. After 90 min, the mixture was evacuated in an Erlenmeyer flask connected to a vacuum pump with 8 mbar of residual pressure for 90 min and at a reduced mixing speed (50 rpm). Next, 7.5 g of hardener (W 241 NF) was added to the mixture, and the mixture was stirred at 1500 rpm for 5 min to improve the activating effect of the hardener. Finally, the mixture was vacuumed at a reduced mixing speed (50 rpm) for 5 min and then poured into molds with the following dimensions: (i) Flat square samples with sides of 100 ± 1 mm and a thickness of 2.0 ± 0.3 mm and (ii) dog-bone samples with dimensions according to ISO 527-2 [37]. The manufactured samples were cured in the molds for 168 h at ambient temperature. According to a study by Corcione et al. [38] in which cold-curing epoxy resins have also been investigated, 168 h is considered sufficient for matrix cross-linking.

## 3. Measuring Methods

Considering the planned applications, the thermal and dielectric properties of the manufactured composites were particularly tested. However, the structural and tensile properties were also analyzed to give a more complete view of the manufactured composite properties. All methods used for characterization of the EP resin, as well as the EP/HNT composites, are defined in more detail in the following paragraphs.

### 3.1. Scanning Electron Microscopy

**Figure 1:**
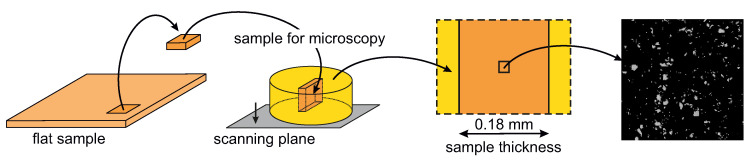
Graphical schema showing the sample preparation for SEM analysis.

The morphology of the prepared composites at the microscopic level was investigated using scanning electron microscopy (SEM). A desktop electron microscope Phenom ProX (Thermo Fisher Scientific, Breda, The Netherlands) was used in environmental mode, i.e., on samples without plating, and the accelerating voltage was set to 15 kV. SEM measurements were carried out in the cross-section plane, which was perpendicular to the largest surface area, as shown in Figure 1 Prior to the analysis, the surface of the samples was thoroughly ground and polished to maximize the SEM image quality.

### 3.2. Simultaneous Thermal Analysis

Simultaneous thermal analysis (STA) is a thermal analysis that can be used to obtain a weight analysis (thermogravimetry, TG) of the analyzed sample and the heat flow through the sample (differential scanning calorimetry, DSC) simultaneously during a controlled temperature program. A simultaneous thermal analyzer (SDT Q600 analyzer, TA Instruments, New Castle, DE, USA) was used to obtain a complex overview of the behavior of tested materials during thermal decomposition. Measurements were carried out with a sample weight of 9.0 ± 0.2 mg (one piece of bulk material in a cuboid shape) and in an air atmosphere with a volume flow rate of 100 mL/min. A uniform temperature interval from 25 ${}^{\circ }$C to 700 ${}^{\circ }$C and a heating rate of 10 ${}^{\circ }$C/min were chosen. Three independent measurements were performed via STA, and the mean values are presented in the results section.

### 3.3. Differential Scanning Calorimetry

The material behavior in the glass transition region was analyzed via DSC using a DSC Q2000 analyzer (TA Instruments, New Castle, DE, USA), which provides better measurement sensitivity within the required temperature range when compared with STA. In all cases, the samples of unfilled EP resin and EP/HNT composites were first heated to 180 ${}^{\circ }$C with a rate of 5 ${}^{\circ }$C/min, then the samples were cooled to 0 ${}^{\circ }$C and reheated again to 180 ${}^{\circ }$C at the same heating rate. This cooling and heating procedure was adopted to ensure the complete curing of the EP resin and to remove its thermal history. All analyses were carried out in a nitrogen atmosphere with a sample weight of 9.0 ± 0.1 mg.

### 3.4. Measurement of Volume Resistivity

The volume resistivity $\rho_{v}$ (Ω·m) is a critical dielectric parameter and shows the current response of the dielectric material when exposed to an external DC field. All measurements were performed according to standard IEC 62631-3-1 [39] in a 500 V DC field and at ambient temperature. The steady-state current was always recorded at the 3600th second. Measurements were carried out with a Keithley 6517A electrometer and Keithley 8009 electrode system with an active surface area of 22.9 cm^2^ (both Keithley Instruments, Cleveland, OH, USA). The polarization index values *PI*_1_ (-) and *PI*_10_ (-), which provide information about the changes in accumulated charge during the polarization processes, were calculated from the current curves at predefined times. *PI*_1_ represents the ratio of total current at the 15th and 60th second (*I*_15_/*I*_60_), while *PI*_10_ is the ratio of total current at the 60th and 600th second (*I*_60_/*I*_600_).

### 3.5. Broadband Dielectric Spectroscopy

Broadband dielectric spectroscopy (BDS) has been utilized to analyze the material response to an alternating electric field with variable frequency *f* (Hz). The modular measurement system was used with an Alpha A mainframe (Novocontrol Technologies, Montabaur, Germany) and ZGS test interface. The real and imaginary parts of the complex permittivity ϵ* (-) were determined in a frequency range from 0.5 Hz to 1 MHz (for each setpoint measured from highest frequency to lowest), and the test voltage was set to 1 V_RMS_ While the real part of the complex permittivity, the so-called dielectric constant ϵ′ (-), is a measure of sample capacity, the imaginary part, the so-called loss factor ϵ″ (-), is a measure of dielectric losses. The measurement was carried out in a temperature range from −50 ${}^{\circ }$C to 100 ${}^{\circ }$C with a temperature step of 5 ${}^{\circ }$C, and the samples were placed between gold-plated electrodes with a diameter of 30 mm.

### 3.6. Mechanical Tensile Tests

Mechanical properties were studied by tensile testing according to ISO 527. In the case of encapsulating and potting materials, the testing of tensile properties is not absolutely necessary but is generally accepted as a reliable and straightforward method for detecting changes in mechanical properties induced by the filling of polymer material. The main observed parameters obtained from tensile tests within this study are stress at yield $\sigma_{y}$ (MPa), strain at yield $\epsilon_{y}$ (%), stress at break $\sigma_{b}$ (MPa), and strain at break $\epsilon_{b}$ (%). Tensile tests were carried out at ambient temperature on a LabTest 3.030 universal testing machine (LaborTech, Opava, Czech Republic) according to the ISO 527 standard using five dog-bone samples (shape 5A) and a recommended test speed of 2 mm/min [37,40]. The stress and strain at yield were evaluated at a point determined by the first local maximum in the deformation curve that appears after a steep increase in stress in the elastic deformation area.

## 4. Results and Discussion

### 4.1. Particle Dispersion Analysis

**Figure 2:**
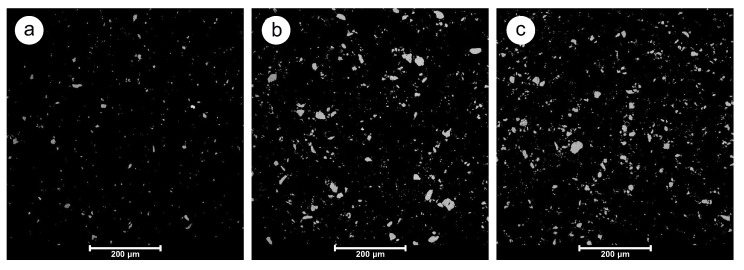
SEM images of the manufactured EP/ HNTs cross-sections: (**a**) EP/2.5HNT, (**b**) EP/6.5HNT, and (**c**) EP/10HNT.

SEM images of the EP/HNTs composite structures with different HNT filler concentrations are shown in Figure 2 Importantly, all images in Figure 2 are presented after additional adjustment of the color scale, brightness, contrast, and gamma correction in graphical software to highlight the HNT particles when the polymer matrix is ideally displayed as a black area.

SEM analysis reveals the presence of agglomerations that vary in extent depending on the concentration of HNT filler. The difference in the dispersion of HNTs between the loading levels of 2.5 and 6.5 wt% is significant, while further increasing the loading level up to 10 wt% no longer has a substantial effect on the dispersion level. In general, a low level of filler dispersion is expected from the very beginning of the experiment, since the clay filler is intentionally used without any surface treatment because surface treatments can negatively affect the dielectric properties [36]. It has already been shown in an earlier study by Polanský et al. [41] that polymer composites based on HNTs without any surface treatment and thus with a high level of agglomeration can still achieve desirable dielectric properties for many applications in electrical engineering. Of course, the level of agglomeration can be reduced by using an additional surface treatment of the HNTs, as mentioned in [42,43]; however, the decrease in agglomeration comes at the cost of a decrease in dielectric properties. Nevertheless, although agglomerates widely occur in the produced composites, Figure 2 shows that their distribution within the volume of the material is uniform and has similar characteristics as in the study by Mo et al. [44]. This fact is due to the relatively high viscosity of the EP resin that was used. Its main component EC 141 NF has a declared viscosity η > 650 mPa·s [45], which prevents sedimentation of the filler particles while curing at ambient temperature.

### 4.2. Thermal Analysis

**Figure 3:**
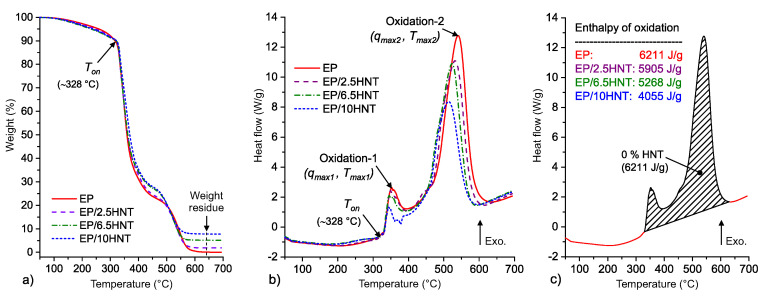
Graphical summary of the simultaneous thermal analysis (STA) results: (**a**) Weight changes (thermogravimetry (TG) curves), (**b**) heat flow changes (differential scanning calorimetry (DSC) curves), and (**c**) example evaluation of the area under the DSC curve for the thermo-oxidation reaction.

Due to the priority of improving the thermal behavior mainly in the case of fire as an extreme emergency condition, a detailed evaluation of the thermal properties of tested materials at high temperatures was conducted. Thermo-oxidation processes accompanied by irreversible changes (primarily degradation) in polymer materials occur at high temperatures, and the data determined by thermal analysis can be used as relevant results [41,46] for parameters defined by standardized methods [27]. The results of the simultaneous measurement of weight (TG curves) and heat flow (DSC curves) are summarized graphically in Figure 3 Only minimal differences are evident from the change in weight for each material (Figure 3a). The most significant weight loss occurs in a temperature range of 320–400 ${}^{\circ }$C, during which a less significant exothermic process (Oxidation-1) in the DSC curves occurs (Figure 3b). More precisely, it is possible to define the beginning of substantial changes in materials associated with thermo-oxidation by the initial temperature *T_on_* (approximately 328 ${}^{\circ }$C for all tested materials—shown in Figure 3b). From an energy point of view, the reduction of the steepness of weight loss occurs at the beginning of the more critical exothermic process (Oxidation-2) for composites with 6.5 and 10 wt% HNTs in comparison with EP and the composite with 2.5 wt% HNTs.

After the thermo-oxidation process (Oxidation-1 and Oxidation-2 regions) is complete at a temperature of approximately 600 ${}^{\circ }$C, different weight residues corresponding to the weight fraction of dehydrated and dehydroxylated HNTs are apparent in Figure 3a. When the temperature of 600 ${}^{\circ }$C is exceeded, all degradation products of the thermo-oxidative decomposition of the EP resin are already burned (discussed in detail in [47]). Hence, there is no significant decrease in HNT weight; furthermore, from the DSC curves and according to [48], only changes in the mineral structure can be assumed to occur during the continued heating of HNTs.

Figure 3b shows that the increase in the concentration of HNT filler causes a decrease in the peak maximum of Oxidation-1 (*T_max_*_1_), whereas this decrease is the most significant at the highest filler level (EP/10HNT). The heat flow local maximum of Oxidation-1 for EP is approximately 1.6 times higher than that for EP/6.5HNT. A similar decreasing trend in peak maximum is also observed in the case of Oxidation-2 (*T_max_*_2_), where a noticeable but not very significant decrease in the maximum temperature occurs. This decrease in temperature *T_max_*_2_ is most likely due to the dehydroxylation of the HNTs, which occurs at a maximum temperature of approximately 482 ${}^{\circ }$C [36] The dehydroxylation of the HNTs slightly shifts the temperature range of the main thermo-oxidation of the polymer matrix (Oxidation-2) with an increasing weight fraction of HNTs and, at the same time, suppresses this intensive process (see a detailed discussion on the barrier effect in [49] and the dilution of degradation products in [50]).

The area under the heat flow curve was further analyzed for all tested composites and neat EP to confirm the positive impact of HNT incorporation on the thermal properties of the polymer matrix. The way in which this analysis was carried out, together with its main results, is summarized in Figure 3c. The area under the DSC curve (indicated by the hatching of the Oxidation-1 and Oxidation-2 temperature intervals) is the largest for pure epoxy resin and the smallest for the composite with 10 wt% HNT, as is evident from Figure 3c. The area under the DSC curve represents the specific enthalpy *h_ox_* (J/g) of all reactions ongoing in tested samples (in EP/HNT composite mainly oxidation related to EP and dehydroxylation related to HNT). The summary of values for all important parameters that characterize the Oxidation-1 (maximal peak values of heat flow *q_max_*_1_ and temperature *T_max_*_1_) and Oxidation-2 (*q_max_*_2_ and *T_max_*_2_) processes together with the calculated values of specific enthalpy *h_ox_* are listed in Table 1.

**Figure 4:**
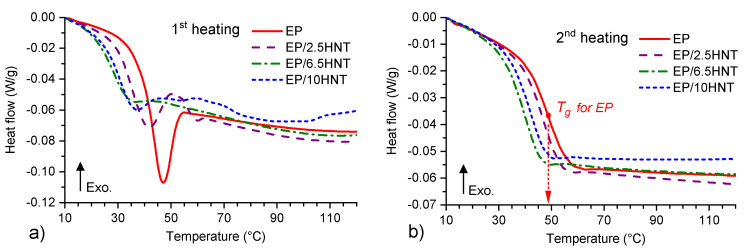
Temperature dependencies of heat flow for (**a**) first heating, and for (**b**) second heating together with highlighted *T_g_* for EP.

In addition to determining the thermal stability of the tested materials at temperatures above 200 ${}^{\circ }$C, their thermal properties under normal operating conditions were also analyzed via a DSC Q2000 analyzer to enhance the measurement sensitivity in the glass transition region. As mentioned above, the materials were reheated from 0 ${}^{\circ }$C to 180 ${}^{\circ }$C during the DSC measurement. Hence, all the effects commonly related to every cured thermosetting system that take place when the resin is exposed to the first notable thermal stress during operation (e.g., post-curing, the release of volatiles, post-dehydration, and others) were significantly reduced during the second heating. This fact was especially true for the resin used in this study, as it was cured only at ambient temperature under standard laboratory conditions. Therefore, the very first thermal stress was that experienced in the DSC furnace during the first heating of the resin. The effect of moisture (described in [51,52]) or other volatile residues was thus minimized, and among other outcomes, an increased level of polymer chain cross-linking was also expected. The *T_g_* values were evaluated as the inflection point of the steep decrease in the DSC curve during the glass transition. The curves of the heat flow during the first and second heating of the test samples are shown in Figure 4, and the obtained *T_g_* values are summarized in Table 1.

At first glance, it is evident from Figure 4a,b that the character of the glass transition region, as well as the absolute values of *T_g_*, have significantly changed when comparing the first and second heating. Furthermore, the *T_g_* values may overall be low (below 50 ${}^{\circ }$C) compared to the other epoxy systems used in electrical engineering [53]. However, when the obtained values are strictly compared to cold-curing epoxy resin systems, very good agreement can be found with the publications from other authors, e.g., [54,55]. The whole system of processes associated with post-curing and the release of residual volatiles during the first heating is clearly visible in Figure 4a, where the typical steep decrease in the DSC baseline during the glass transition is overlapped by enthalpy relaxation [56,57]. The most significant reactions are in the case of the EP resin without HNTs, where the barriers capable of suppressing the post-curing of the EP resin are not present. As the weight fraction of the HNTs in the composite increases, the heat flow changes in the glass transition region are less pronounced.

A comparison of the *T_g_* values summarized in Table 1 indicates a reduction in the glass transition temperature on the order of a few degrees Celsius owing to the addition of HNTs. In this respect, it can be assumed that the presence of HNTs affects the ability of the EP resin to cross-link and likely reduces the interconnection of the polymer chains. Hence, HNTs without a surface modification act primarily as a nonreactive component of the material, and thus, essentially act as a cross-linking inhibitor. An apparent decrease in *T_g_* with the increasing volume fraction of HNTs is visible for EP/2.5HNT and EP/6.5HNT. The opposite trend follows, and *T_g_* increases slightly for the EP/10HNT sample. This behavior may be due to changes in the thermal conductivity of the sample, which is influenced not only by the increase in the HNT volume fraction but also by the presence and mutual interaction of HNT agglomerates. It is clear from the literature [58,59,60,61] that the addition of a mineral filler generally increases the thermal conductivity of an epoxy-based composite and thereby affects other thermal parameters. These changes depend markedly on the chosen polymer (i.e., on the type of epoxy resin), the selected mineral filler, and its surface modification. However, HNTs may form thermally conductive paths that can affect the rate of change in the thermal capacity of the sample in the glass transformation region. In this manner, HNTs may shift the moment of heat capacity change in the sample to higher temperatures; thus, the *T_g_* values slightly increase again, as shown in the EP/10HNT sample. Nevertheless, the observed differences are small because the addition of HNTs causes only a minimal increase in the thermal conductivity of the epoxy-based material [44].

### 4.3. Dielectric Analysis

As previously mentioned, the main dielectric parameters have been investigated. First, the results obtained from the absorption currents during the application of DC voltage are presented in Table 2.

The Table 2 summarizes the mean values for the volume resistivity and polarization index along with standard deviations from the three measurements. From these values, it is evident that with the higher concentration of filler, the volume resistivity and polarization index values decrease. A significant change in the parameters occurs mainly between the 2.5 and 6.5 wt% HNT concentrations.

The changes in the monitored parameters are most likely related to (i) the increasing incorporation of free ions into the polymer matrix [62]; (ii) the increasing number of agglomerates resulting in a high volume of a polymer-filler interphase, which is connected with the occurrence of interfacial polarizations [63,64]; and (iii) the increasing presence of absorbed water [65]. Moreover, the absorbed water may lead to the breaking of weak bonds in the main polymer chains, which may strongly affect the total number of dipoles in the system [66]. All these aspects can together contribute to the increase in conductive current increasing.

**Figure 5:**
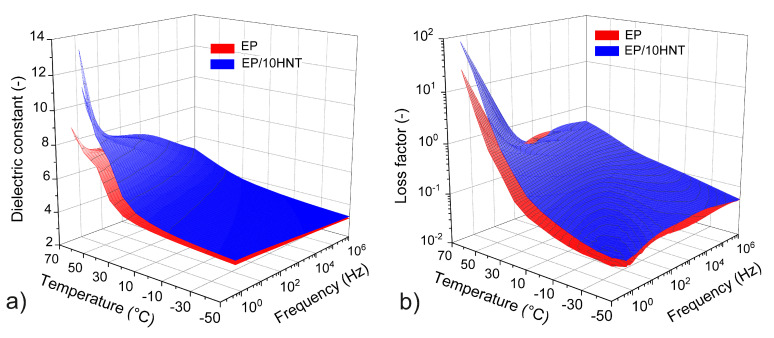
Temperature-frequency dependencies of the (**a**) dielectric constant and (**b**) loss factor for the material based on an epoxy resin and for an epoxy composite with 10 wt% HNTs.

Broadband dielectric spectroscopy was further used to observe the changes in dielectric properties at various temperatures and frequencies of the electrical field, which in turn was used to analyze the dielectric behavior of the tested composites in more detail. Figure 5 shows the temperature-frequency dependence of the dielectric constant and loss factor for EP and EP/10HNT. The ϵ′ and ϵ″ dependencies for composites with filler levels of 2.5 and 6.5 wt% HNTs are omitted for the clarity of the graph; nevertheless, their surfaces are regularly placed between the two surfaces shown and correspond to the increasing trend in both parameters.

It is apparent from the presented dependencies that the change in the dielectric constants and loss factor values are not very significant even at the maximum volume fraction of HNTs tested (10 wt%). Due to the relatively significant polarity of the selected epoxy resin, the addition of a low wt% of HNT does not substantially affect the polarity of the resulting composite. Only at the highest selected temperatures (above 60 ${}^{\circ }$C) and at low frequencies (below 1 Hz) is there a more apparent increase in the dielectric constant and loss factor due to the incorporation of the filler. Other significant polarization mechanisms are not visible from the presented results because DC conductivity and Maxwell−Wagner−Sillars (MWS) polarization generally overlap the individual interfacial contributions [67], which usually occur at low frequencies and high temperatures. However, the behavior of the tested materials corresponds to the observed changes in the absorption currents mentioned above; thus, the area of low frequencies (including DC) may indicate a potential deterioration of dielectric properties as well. Another reason for the increase in the investigated parameters may be that the incorporated HNTs have a relatively high dielectric constant, which is also dependent on the frequency and temperature (ϵ′ > 10 [36]).

**Figure 6:**
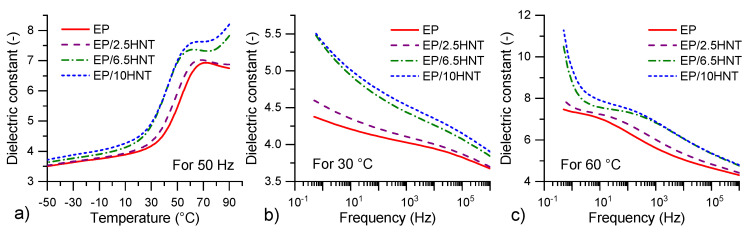
Dielectric constant characteristics: (**a**) Temperature dependencies at 50 Hz, (**b**) frequency dependencies at 30 ${}^{\circ }$C, and (**c**) frequency dependencies at 60 ${}^{\circ }$C for all tested materials.

**Figure 7:**
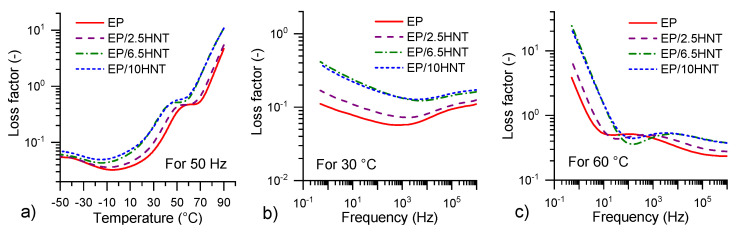
Loss factor characteristics: (**a**) Temperature dependencies at 50 Hz, (**b**) frequency dependencies at 30 ${}^{\circ }$C, and (**c**) frequency dependencies at 60 ${}^{\circ }$C for all tested materials.

Figure 6 and Figure 7 show the critical frequency and temperature dependencies of the dielectric constant and loss factor as a curve extracted from Figure 5 These curves are selected for a more detailed comparison under possible industrial operating conditions. There is a more easily visible shift in individual maximum peak values toward lower temperatures and frequencies, depending on the HNT content. This is most likely due to the combination of structural changes leading to (i) the gradual growth of permanent dipoles, which contribute to the shift in dipolar polarization and increase its intensity [41,64,68], and (ii) the curing ability inhibition of EP/HNT composites, which has been demonstrated via the DSC measurements. However, from these figures, it is also visible that MWS polarizations and DC conductivity cause the most dominant dielectric responses in the tested EP/HNT composites.

### 4.4. Evaluation of Mechanical Tensile Tests

The main parameters derived from the tensile tests of the tested EP resin and EP/HNTs are summarized in Table 3 At first glance, it is evident that the increasing volume fraction of HNTs in the tested composites results in a reduced tensile strength, as demonstrated by a gradual decrease in the parameters of stress at yield and stress at break for composites with 6.5 and 10 wt% HNTs. This behavior can be caused by the presence of HNT agglomerates (Figure 1), which can represent stress concentrators [69] due to less interfacial bonding between the HNTs and polymer matrix, as has been already observed by Razak et al. [70] and Wang et al. [71].

Regarding this fact, the purity of the particles could also be taken into account. Used particles contain ≈84% of HNTs, and rest are impurities (kaolinite, quartz, gibbsite, and alunite) [35]. In our case, additional surface treatment or HNTs decontamination [72] have not been performed, and the presence of kaolinite with plate structure [73] in non-exfoliated form could partially contribute to this behavior. There is an assumption that a different behavior could be achieved by the purification of used particles; however, it was not the primary goal of presented experiment.

This behavior can be also attributed to the heterogeneous density of the nanocomposite and the presence of weak boundaries between particles and trapped air during the sample preparation, according to the study of Vahedi et al. [74]. It is also necessary to mention that the tested epoxy resin is a fairly complex chemical structure since it is cured at ambient temperature using a polyamine hardener; thus, there may also be other interactions [75]. However, it is generally agreed that polymer materials incorporating HNTs are tough in terms of their mechanical properties [74,76]. Therefore, the incorporation of HNTs reinforces the epoxy according to the theory; however, HNTs inhibit cross-linking, so the material becomes more flexible instead of more rigid, which can be useful in specific applications (e.g., for equipment exposed to vibrations and occasional minor impacts to the protective insulation layer [77]).

## 5. Conclusions

Cold-curing epoxy-based composites with various concentrations of HNT filler were tested to mainly evaluate their fire retardancy, dielectric, and mechanical properties. Even though the tested composites were manufactured under laboratory conditions, the way they were prepared was chosen in full accordance with their possible use in practice, which results in the formation of uniformly dispersed agglomerates. The DSC results show a decrease in the heat flow maximum of the primary thermo-oxidation process and a decrease in the specific enthalpy. Furthermore, as also observed from DSC, the glass transition temperature decreases by several degrees Celsius with increasing concentrations of HNT filler up to 6.5% HNTs. For the highest filler concentration, the limitation of the glass transition temperature trend is apparent. The dielectric properties of the manufactured EP/HNT composites were analyzed via BDS, which proved that there is a slightly increasing trend in the values of the dielectric constant and an insignificant difference in the values of the loss factor as a measure of dielectric losses. A slight decrease in volume resistivity with increasing concentrations of HNT filler is also observed. The addition of HNTs into the EP matrix also affects the mechanical properties of the resulting composite, where the tensile stress at break significantly decreases, and the strain at break increases.

## Figures and Tables

**Table 1:** Summary of the main parameters derived from the DSC thermograms obtained by various analyses.

Material			SDT Q600			DSC Q2000
	*q_max_*_1_ (W/g)	*T_max_*_1_ (${}^{\circ }$C)	*q_max_*_2_ (W/g)	*T_max_*_2_ (${}^{\circ }$C)	*h_ox_* (J/g)	*T_g_* (${}^{\circ }$C)
EP	2.63	352.5	12.77	540.3	6211	52.71
EP/2.5HNT	2.40	348.3	11.09	531.6	5905	47.92
EP/6.5HNT	2.11	344.0	10.90	523.2	5268	43.50
EP/10HNT	1.33	343.2	8.37	513.9	4055	44.61

**Table 2:** Dielectric parameters estimated from the absorption current.

Material	$\rho_{v}$ (Ω·m)	*PI*_1_ (-)	*PI*_10_ (-)
EP	(1.70 ± 0.7) $\times 10^{15}$	2.66 ± 0.1	3.87 ± 0.4
EP/2.5HNT	(1.02 ± 0.2) $\times 10^{15}$	2.64 ± 0.1	3.93 ± 0.1
EP/6.5HNT	(1.4 ± 0.1) $\times 10^{14}$	2.28 ± 0.1	2.68 ± 0.1
EP/10HNT	(2.0 ± 0.4) $\times 10^{14}$	2.32 ± 0.1	2.97 ± 0.2

**Table 3:** Summary of the main parameters derived from the tensile tests of the EP resin and EP/HNTs.

Material	Stress at Yield (MPa)	Stress at Break (MPa)	Strain at Yield (%)	Strain at Break (%)
EP	52.42 ± 4.6	52.39 ± 4.6	5.62 ± 0.6	5.62 ± 0.6
EP/2.5HNT	40.74 ± 3.9	40.04 ± 3.3	4.66 ± 0.5	4.71 ± 0.6
EP/6.5HNT	21.69 ± 3.4	12.79 ± 3.5	5.39 ± 0.3	27.44 ± 13.3
EP/10HNT	15.38 ± 2.2	9.80 ± 2.9	5.52 ± 0.4	34.56 ± 12.2
